# Stemness-Associated Markers Are Expressed in Extracranial Arteriovenous Malformation

**DOI:** 10.3389/fsurg.2021.621089

**Published:** 2021-03-19

**Authors:** Claire S. Luke Krishnan, Helen D. Brasch, Josie Patel, Nicholas Bockett, Erin Paterson, Paul F. Davis, Swee T. Tan

**Affiliations:** ^1^Gillies McIndoe Research Institute, Wellington, New Zealand; ^2^Centre for the Study & Treatment of Vascular Birthmarks, Wellington Regional Plastic, Maxillofacial and Burns Unit, Hutt Hospital, Wellington, New Zealand; ^3^Department of Surgery, The Royal Melbourne Hospital, The University of Melbourne, Melbourne, VIC, Australia

**Keywords:** vascular malformation, arteriovenous malformation, embryonic stem cells, induced pluripotent stem cell, somatic activating mutations, stemness-associated markers

## Abstract

**Objectives:** Arteriovenous malformation (AVM) consists of a *nidus* with poorly formed low-resistance vessels in place of a functional capillary network. The role of somatic mutations in embryonic stem cells (ESCs) and vascular anomalies and the presence of primitive populations in vascular anomalies led us to investigate the presence of a primitive population in extracranial AVM.

**Methods:** Extracranial AVM tissue samples from 12 patients were stained for stemness-associated markers OCT4, SOX2, NANOG, KLF4, and c-MYC using immunohistochemical staining. *In situ* hybridization (ISH) was performed on six tissue samples to determine transcript expression. Western blotting and RT-qPCR were performed on two AVM-derived primary cell lines to determine protein and transcript expression of these markers, respectively. Immunofluorescence staining was performed on two tissue samples to investigate marker co-localization.

**Results:** Immunohistochemical staining demonstrated the expression of OCT4, SOX2, KLF4, and c-MYC on the endothelium and media of lesional vessels and cells within the stroma of the *nidus* in all 12 AVM tissue samples. ISH and RT-qPCR confirmed transcript expression of all five markers. Western blotting showed protein expression of all markers except NANOG. Immunofluorescence staining demonstrated an OCT4+/SOX2+/KLF4+/c-MYC+ population within the endothelium and media of the lesional vessels and cells within the stroma of the AVM *nidus*.

**Conclusions:** Our findings may suggest the presence of a primitive population within the AVM *nidus*. Further investigation may lead to novel therapeutic targeting of this population.

## Introduction

Extracranial arteriovenous malformation (AVM) is a rare (1), non-heritable condition (2) with no sex or racial predilection (3). This high-flow vascular malformation (1, 4) is present at birth and progresses during adolescence or adulthood (1, 3). AVM is characterized by a central *nidus*, consisting of poorly formed vessels with direct connections between the high-flow arteries and low-flow veins, in place of normal capillaries (5) causing arteriovenous shunting (6). Histologically, AVM has a fibrous background underlying tortuous small vessels intermingled with thick-walled larger vessels with luminal dilation (5, 7, 8). Although predominantly affecting the head and neck, AVM can occur anywhere in the body. It is diagnosed by careful history and examination and confirmed by ultrasonography to identify arteriovenous shunting (9), magnetic resonance, and/or superselective angiography (10), demonstrating arterial feeders and draining vein(s) (1).

Standard treatment for AVM is surgical excision of the *nidus* and reconstruction following preoperative embolization (7), as required. Yakes (11) reports the efficacy of direct ethanol injection into the *nidus*, with ethanol embolization of arterial feeders and outflow vein(s) (12). Ethanol destroys the endothelium and induces thrombosis in the channels, preventing neovascular recruitment, or recanalization and minimizing risk of recurrence (11). Management of AVM remains challenging with a high incidence of recurrence (3).

The pathogenesis of AVM remains unclear and may result from an embryonic vascular developmental error (1). A somatic activating mutation in the *MAP2K1* gene causing endothelial dysfunction *via* increased MEK1 activity has been proposed (2, 13). Extracellular diffusion of von Willebrand factor (vWF) and staining on the pericellular rim in AVM has demonstrated endothelial cell dysfunction and leakage (14). Endothelial localization of the mutation correlates with severe phenotypic alterations in blood vessels of mutated mice (2, 15).

Embryonic stem cells (ESCs) originate from the inner cell mass of the blastocyst (16) and express transcription factors (17) such as octamer-binding protein 4 (OCT4), sex-determining region Y-box 2 (SOX2), homeobox transcription factor NANOG, Krüppel-like factor 4 (KLF4), and proto-oncogene c-MYC, all of which maintain an ESC phenotype by regulating pluripotency and self-renewal (16, 18, 19).

Takahashi et al. have demonstrated induction of pluripotent stem cells from adult mouse (17) and human (20) fibroblasts by introducing the transcription factors OCT4, SOX2, c-MYC, and KLF4. Yu et al. (21) also achieve this with NANOG and LIN28 in place of c-MYC and KLF4, underscoring the sufficiency of these four transcription factors (17) to generate induced pluripotent stem cells (iPSCs).

OCT4, SOX2, and NANOG confer ESC identity through specification of ESC fate and self-renewal properties (22). OCT4 and SOX2 collaboratively induce nuclear transcription of target genes such as NANOG (23). Interestingly, NANOG-deficient iPSCs are transcriptionally similar to wild-type iPSCs (24). KLF4 additionally maintains vascular integrity during embryogenesis and reprograms somatic cells into iPSCs (25). c-MYC maintains cellular proliferation, growth, and survival by controlling cell cycle regulator expression (26). In this manuscript, these markers are referred to as stemness-associated markers.

The presence and, in particular, the patterns of expression of stemness-associated markers in vascular anomalies suggest a role for stem cells in their pathogenesis. Endothelial expression of stem cell marker CD133 has been demonstrated in vessels and stromal cells of lymphatic malformation, and *NANOG* and *OCT4* genes are present within CD133+ patient-derived cells (27).

We have previously demonstrated the presence of an ESC-like population in vascular tumors (28, 29) and vascular malformations (22, 30, 31). This study investigated the presence and expression pattern of OCT4, SOX2, NANOG, KLF4, and c-MYC in extracranial AVM, using immunohistochemical and immunofluorescence staining, *in situ* hybridization (ISH), reverse transcription quantitative polymerase chain reaction (RT-qPCR), and western blotting (WB).

## Materials and Methods

### Tissue Samples

Extracranial AVM tissue samples from six male and six female patients, aged 17–65 (mean, 34.4) years (Supplementary Table 1), were sourced from the Gillies McIndoe Research Institute Tissue Bank. This study was approved by the Central Health and Disability Ethics Committee (Ref. 13/CEN/130), with written informed consent from all participants.

### AVM-Derived Primary Cell Lines

Primary cell lines were derived from two fresh AVM tissue samples from the original cohort of 12 patients. Samples were cut into small pieces and incubated between layers of Matrigel (cat#354234, Corning Life Sciences, Tewksbury, MA, USA) in a 24-well-plate with a media containing Dulbecco's modified Eagle's medium (DMEM) with GlutaMax^TM^ (cat#10569010, Gibco, Rockford, IL, USA), supplemented with 2% penicillin–streptomycin (cat#15140122, Gibco), and 0.2% gentamicin/amphotericin B (cat#R01510, Gibco). Once sufficient cell growth was achieved to support transfer to a monolayer culture, cells were extracted by dissolving the Matrigel with Dispase (cat#354235, Corning Life Sciences) and transferred to an adherent culture flask with media containing DMEM with GlutaMAX™ supplemented with 10% fetal bovine serum (cat#10091148, Gibco), 5% mTeSR^TM^ 1 Complete Medium (cat#E8580, STEMCELL Technologies, Vancouver, BC, Canada), 1% penicillin–streptomycin (cat#15140122, Gibco), and 0.2% gentamicin/amphotericin B (cat#R01510, Gibco) at 37°C and 5% CO_2_. Cells were expanded in culture and harvested at passages 5 and 6.

### Histochemical, Immunohistochemical, and Immunofluorescence Staining

Hematoxylin and eosin (H&E) staining was performed on 4-μm-thick consecutive formalin-fixed paraffin-embedded (FFPE) sections of 12 extracranial AVM tissue samples, to confirm the presence of AVM by an anatomical pathologist. Immunohistochemical staining of these tissue sections was performed on the Leica BOND^TM^ RX auto-stainer (Leica, Nussloch, Germany) with primary antibodies for OCT4 (1:30; cat#309M-16, Cell Marque, Rocklin, CA, USA), SOX2 (1:500; cat#PA1-094; ThermoFisher), NANOG (1:200; cat#EP225; Cell Marque), KLF4 (1:100; cat#NBP2-24749, Novus Biologicals, Centennial, CO, USA), and c-MYC (1:1,000; cat#ab32, Abcam, Cambridge, MA, USA) using the BOND^TM^ Polymer Refine Detection kit (cat#DS9800, Leica), using 3,3′-diaminobenzidine as the chromogen. Immunohistochemical-stained slides were mounted in Dako Mounting Medium (cat#CS703, Dako, Glostrup, Denmark). Positive human control tissues were seminoma (OCT4 and NANOG), skin (SOX2), breast carcinoma (KLF4), and normal colon (c-MYC). Negative controls were AVM sections run with mouse (ready-to-use; cat#IR750, Dako) or rabbit (ready-to-use; cat#IR600, Dako) isotype controls.

To confirm the co-expression of two proteins, immunofluorescence staining was performed on two AVM tissue samples, using the same primary antibodies with the same concentrations, and co-staining with endothelial marker VWF (1:200; cat#A0082, Dako) and smooth muscle actin (SMA; ready-to-use; cat#PA0943, Leica), to identify endothelium and smooth muscle within the *nidus*, respectively. Appropriate secondary antibodies and amplification kits were used for immunofluorescence detection: VectaFluor Excel anti-rabbit 594 (ready-to-use; cat#DK-1594, Vector Laboratories, Burlingame, CA, USA) using Alexa Fluor 488 (1:500; cat#A21202, Invitrogen) donkey anti-mouse as a secondary and anti-mouse 488 (ready-to-use; cat#DK-2488, Vector Laboratories) using Alexa Fluor 594 (1:500; cat#A21207, Invitrogen) donkey anti-rabbit as a secondary. KLF4, NANOG, and SOX2 were co-stained with endothelial marker CD34 (ready-to-use; cat#PA0212, Leica). All antibodies were diluted with BOND primary antibody diluents (cat#AR9352, Leica). Slides were mounted using Vectashield hardset medium with 4′,6-diamidino-2-phenylindone (cat#H-1500, Vector Laboratories).

Negative controls for immunofluorescence staining were performed on AVM sections using primary isotype mouse (ready-to-use; cat#IR750, Dako) and rabbit (ready-to-use; cat#IR600, Dako) isotype controls. All immunofluorescence staining was performed on the Leica BOND^TM^ RX auto-stainer using a BOND Detection system (cat#DS9455, Leica).

### Image Capture and Analysis

Immunohistochemical-stained slides were viewed and imaged on the Olympus BX53 light microscope with an Olympus SC100 camera (Olympus, Tokyo, Japan) and processed with cellSens 2.0 software (Olympus). Immunofluorescence-stained slides were viewed and imaged with an Olympus FV1200 biological confocal laser-scanning microscope and processed with cellSens Dimension 1.17 software (Olympus).

### *In situ* Hybridization

Four-micrometer-thick FFPE sections of six AVM tissue samples underwent ISH to confirm the presence of transcripts of stemness-associated marker, using the Leica BOND^TM^ RX auto-stainer (Leica) with probes for OCT4 (cat#592868), SOX2 (cat#477658), KLF4 (cat#457468), and c-MYC (cat#311768). Probes were obtained from Advanced Cell Diagnostics (Newark, CA, USA) and detected using the RNAscope 2.5 LSx Reagent Brown Kit (cat#322700; Advanced Cell Diagnostics). Positive human control tissues were seminoma (OCT4), malignant melanoma (SOX2), breast carcinoma (KLF4), and normal colon (c-MYC), using RNAscope 2.5LS positive control probe UBC (cat#312028, Advanced Cell Diagnostics). Negative controls were demonstrated on AVM tissue using a probe for the bacterial gene DapB (cat#312038, Advanced Cell Diagnostics).

### RT-qPCR

Total RNA was isolated from two AVM-derived primary cell lines. From frozen cell pellets of 5 × 10^5^ viable cells, total RNA was extracted using the RNeasy Micro Kit (cat#74004, Qiagen, Hilden, Germany). An on-column DNAse digest (cat#79254, Qiagen) step was included to remove potentially contaminating genomic DNA. RNA quantity was determined using a NanoDrop2000 Spectrophotometer (ThermoFisher). Transcriptional expression was analyzed in triplicate using the Rotor-Gene Q (Qiagen), Rotor-Gene Multiplex RT-PCR kit (cat#204974, Qiagen), and TaqMan Gene Expression Assay primer probes on 40 ng of RNA. Primer probes used were OCT4 (Hs03005111_g1), SOX2 (Hs01053049_s1), NANOG (Hs02387400_g1), KLF4 (Hs00358836_m1), and c-MYC (Hs00153408_m1) (cat#4331182, ThermoFisher). Gene expression was normalized to the reference genes GAPDH (Hs99999905_m1), PUM1 (Hs00206469_m1), and PSMB4 (Hs00160598_m1) (cat#4331182, ThermoFisher). Universal human reference RNA (UHR; cat#CLT636690, Takara, Shiga, Japan), total RNA extracted from a range of healthy adult human tissues, was used as the calibrator for the 2^−ΔΔCt^ analysis. Nuclease-free water was added for the no template and no reverse transcriptase controls, and RNA from NTERA2 cells was used as the positive control. The presence of the correctly sized bands from the endpoint amplification products was confirmed using 2% agarose gel electrophoresis (cat#G402002, ThermoFisher) and imaged using the ChemiDoc MP (Bio-Rad, Hercules, CA, USA). Graphs were generated using GraphPad Prism (v8.0.2, Windows, San Diego, CA, USA) and results expressed as fold change relative to UHR. A fold change cutoff was set at 2.0 for upregulated and 0.5 for downregulated genes.

### Western Blotting

Total protein was extracted from two AVM-derived primary cell lines in RIPA buffer (cat#89900; Pierce Biotechnology, Rockford, IL, USA). Protein was quantified using a BCA assay (cat#23227, Pierce Biotechnology) and diluted in an equal volume of 2× LDS (cat#B0007, Invitrogen, Carlsbad, CA, USA). Twenty micrograms of total protein was separated by SDS-PAGE on 4–12% Bis-Tris gels (cat#NW04122BOX, Invitrogen) in MES SDS running buffer (cat#B0002, Invitrogen) and transferred to a PVDF membrane (cat#IB24001, Invitrogen) using an iBlot 2 (cat#IB21001, ThermoFisher). Protein was detected on the iBind Flex (cat#SLF2000, ThermoFisher) using primary antibodies for OCT4 (1:1,000; cat#ab109183, Abcam), SOX2 (1:500; cat#48-1400, ThermoFisher), NANOG (1:500; cat#ab109250, Abcam), KLF4 (1:1,000; cat#NBP2-24749, Novus Biologicals), c-MYC (1:1,000; cat#ab32072, Abcam), and α-tubulin (1:2,000; cat#62204, ThermoFisher). Secondary antibodies used were goat anti-rabbit IgG-HRP (1:1,000; cat#ab6721, Abcam) for stemness-associated markers and goat anti-mouse Alexa Fluor 488 (1:1,000; cat#A21202, Life Technologies, Carlsbad, CA, USA) for α-tubulin. Positive control was the NTERA2 human cell line. To visualize HRP protein bands, Clarity Western ECL substrate (cat#1705061, Bio-Rad) was used with the ChemiDocMP Imaging System (Bio-Rad) and Image Lab 6.0 software (Bio-Rad) to analyze protein bands.

## Results

### AVM Tissue Samples Showed the Characteristic *Nidus*

**Figure 1:**
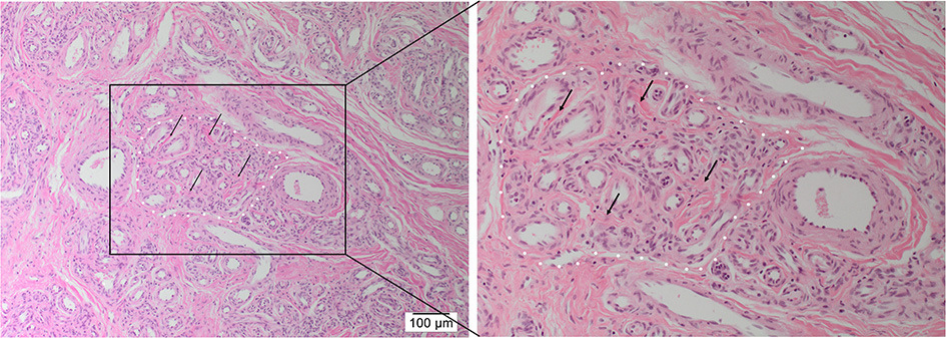
Representative hematoxylin and eosin-stained section of arteriovenous malformation showing the *nidus* (*white dotted outline*) consisting of a tangle of tortuous and dilated thick-walled vessels separated by a fibromyxomatous stroma (*arrows*). Original magnifications: ×100 (magnified view: ×200).

H&E staining demonstrated the characteristic *nidus* consisting of tortuous small vessels surrounded by arterioles and venules, intermingled with thickened arteries and dilated veins on a fibrous background (8) in 12 AVM tissue samples (Figure 1).

### AVM Tissue Samples Expressed OCT4, SOX2, KLF4, and c-MYC Proteins

**Figure 2:**
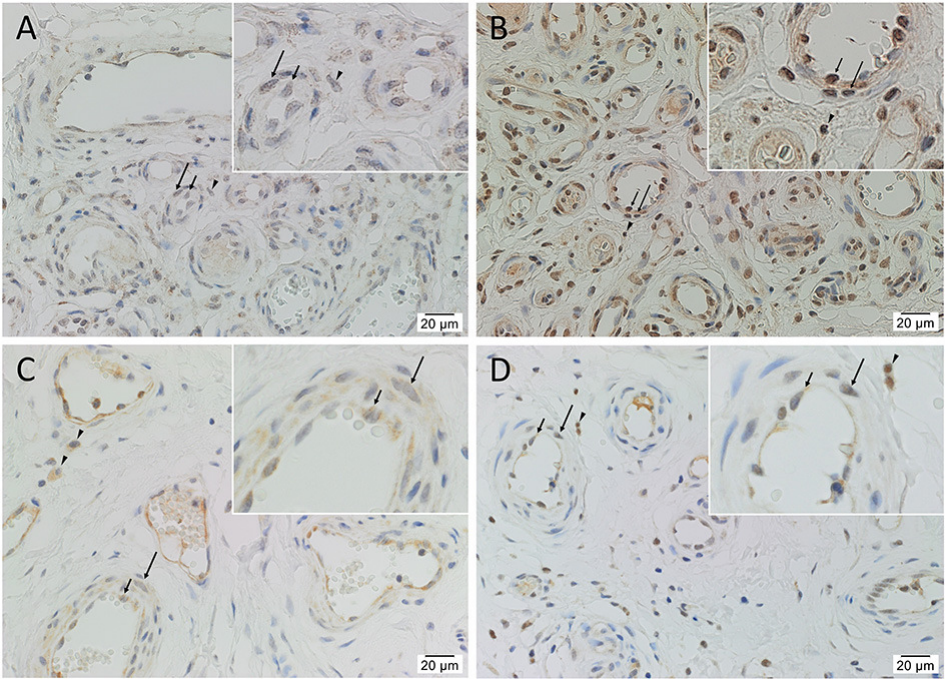
Representative immunohistochemical-stained sections of arteriovenous malformation tissue samples demonstrating the expression of OCT4 (**A**, brown) and SOX2 (**B**, brown), KLF4 (**C**, brown), and c-MYC (**D**, brown) on the endothelium (*short arrows*) and the media (*long arrows*) of the lesional vessels and on cells within the stroma (*arrowheads*) of the *nidus*. Nuclei were counterstained with hematoxylin (**A–D**, blue). Original magnification: ×400.

Immunohistochemical staining demonstrated nuclear and cytoplasmic expression of OCT4 on the endothelium and media of lesional vessels, with nuclear staining of cells within the stroma of the *nidus* (Figure 2A), in all tissue samples. Nuclear and cytoplasmic expression of SOX2 and KLF4 and weaker nuclear expression of c-MYC were demonstrated on the endothelium and media of lesional vessels, with some staining on cells within the stroma of the *nidus* (Figures 2B,D) in all samples. NANOG was not expressed (Supplementary Figure 1) in any of the samples. Magnified figure insets have been provided to show enlarged views of the corresponding images.

Positive controls showed expected staining for OCT4 (Supplementary Figure 2A) and NANOG (Supplementary Figure 2C) in seminoma, SOX2 (Supplementary Figure 2B) in skin, KLF4 (Supplementary Figure 2D) in breast carcinoma, and c-MYC (Supplementary Figure 2E) in normal colon. Immunohistochemical staining of AVM tissue with primary antibodies omitted showed no staining (Supplementary Figure 2F).

### An OCT4+/SOX2+/KLF4+/c-MYC+ Population Was Present on the Endothelium and Media of the Lesional Vessels and Stroma of the *Nidus* of AVM

**Figure 3:**
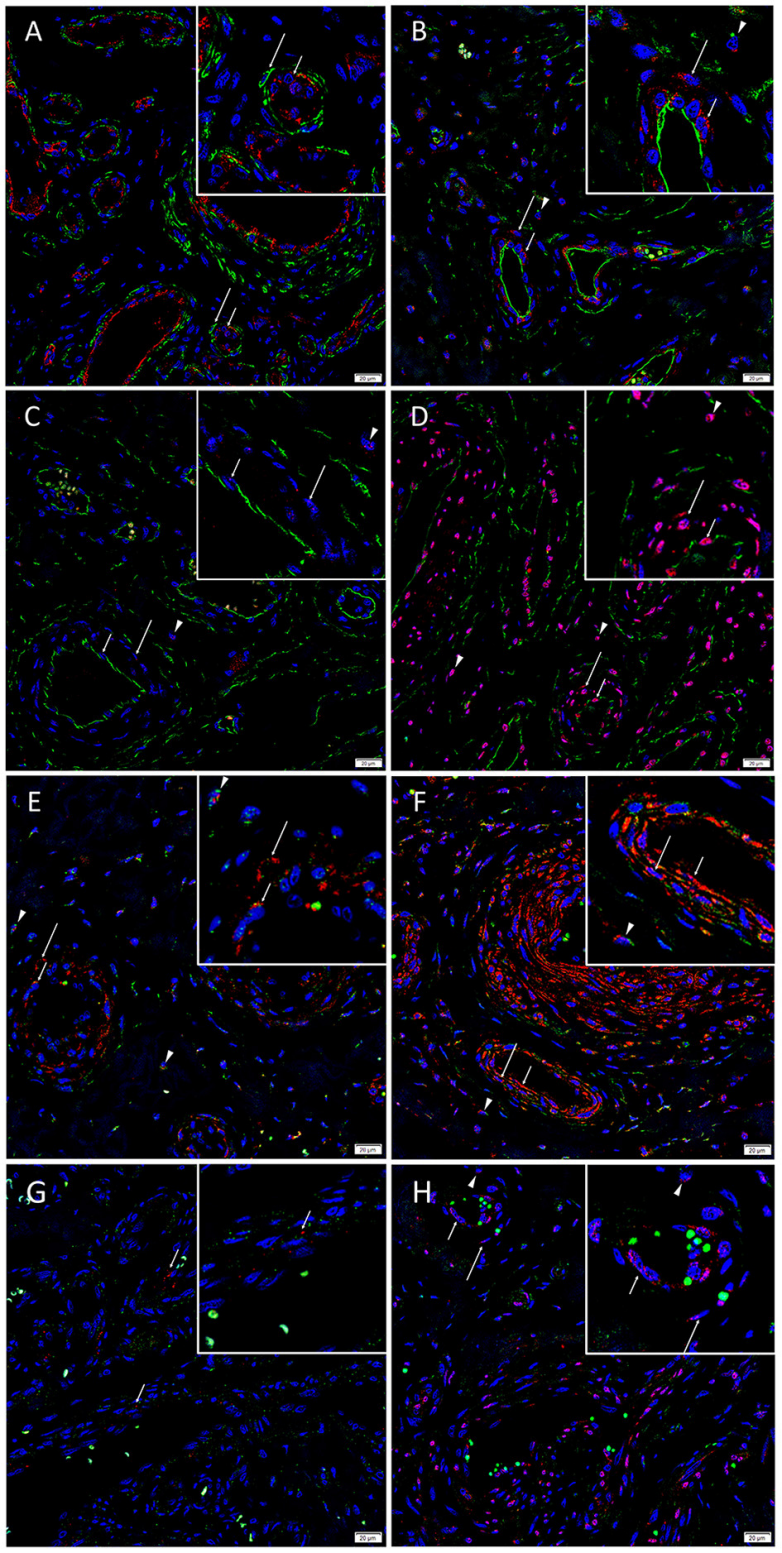
Representative immunofluorescence-stained sections of arteriovenous malformation tissue samples demonstrating endothelial staining of vWF (**A**, red) surrounded by the SMA+ (**A**, green) media of the lesional vessels. The CD34+ endothelium (**B**, green, *short arrows*) and media (*long arrows*) of lesional vessels and the cells within the stroma (*arrowheads*) expressed KLF4 (**B**, red). NANOG (**C**, red) was minimally expressed on the CD34+ endothelium (**C**, green, *short arrows*), media (*long arrows*), and cells within the stroma (*arrowheads*). SOX2 (**D**, red) was expressed on the CD34+ endothelium (**D**, green, *short arrows*) with nuclear staining in the media (*long arrows*) and on cells within the stroma (*arrowheads*). Dual staining of KLF4 (**E,F**, red) with OCT4 (**E**, green) and c-MYC (**F**, green) confirmed the co-expression of KLF4 with both markers. NANOG (**G**, red, *short arrows*) was expressed on some OCT4+ (**G**, green) endothelium of the lesional vessels. SOX2 (**H**, red) and c-MYC (**H**, green) were co-expressed on the endothelium (*short arrows*) and media (*long arrows*) and on cells within the stroma (*arrowheads*). Cell nuclei were counterstained with 4′,6-diamidino-2-phenylindone (**A–H**, blue). Original magnification: ×400. The insets show enlarged views of the corresponding images.

Dual immunofluorescence staining of vWF and SMA demonstrated vessel architecture with a vWF+ inner endothelium (Figure 3A, red) and SMA+ outer media (Figure 3A, green). The CD34+ endothelium (Figure 3B, green) and media of lesional vessels and cells within the stroma of the *nidus* expressed KLF4 (Figure 3B, red). NANOG (Figure 3C, red) was minimally expressed on the CD34+ endothelium (Figure 3C, green) and media and cells within the stroma of the *nidus*. SOX2 (Figure 3D, red) was expressed on the CD34+ endothelium (Figure 3D, green) with nuclear staining in the media and in cells within the stroma of the *nidus*. Dual staining of KLF4 (Figures 3E,F, red) with OCT4 (Figure 3E, green) and c-MYC (Figure 3F, green) confirmed the co-expression of KLF4 with both markers. NANOG (Figure 3G, red) was expressed on some OCT4+ (Figure 3G, green) endothelium of the lesional vessels. SOX2 (Figure 3H, red) and c-MYC (Figure 3H, green) were co-expressed on the endothelium and media and in cells within the stroma of the *nidus*. Magnified figure insets have been provided to show enlarged views of the corresponding images.

Supplementary Figure 3 presents split images of stains in Figure 3. Negative control demonstrated no staining (Supplementary Figure 3Q), confirming primary antibody specificity.

### AVM Tissue Samples Expressed OCT4, SOX2, KLF4, and c-MYC Transcripts

**Figure 4:**
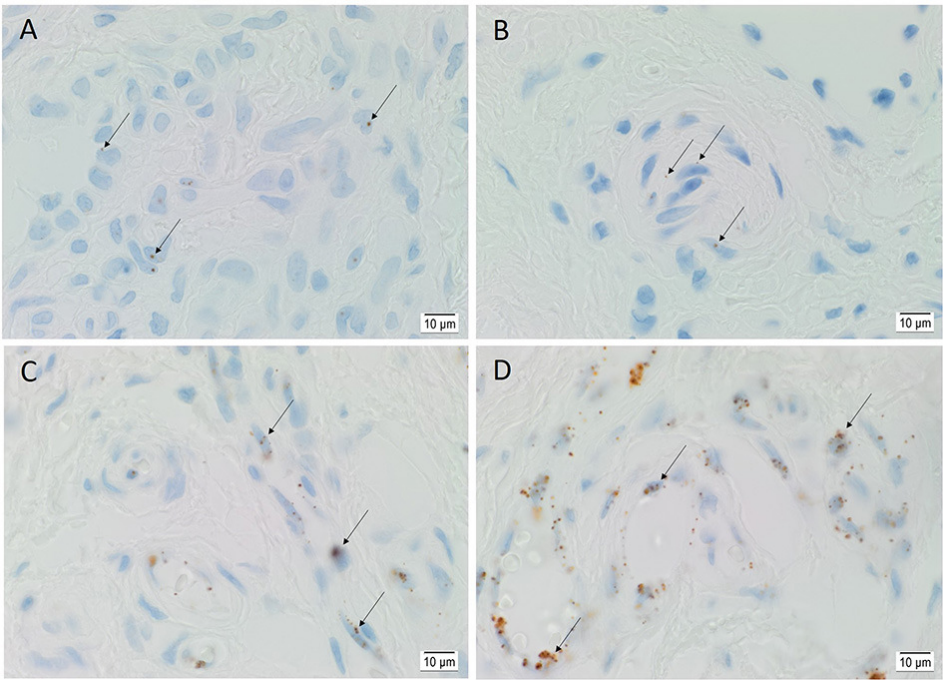
Representative *in situ* hybridization sections of arteriovenous malformation tissue samples demonstrating the presence of transcripts for OCT4 (**A**, brown), SOX2 (**B**, brown), KLF4 (**C**, brown), and c-MYC (**D**, brown) on the endothelium (*long arrows*) of the lesional vessels. OCT4 (**A**, brown), KLF4 (**C**, brown), and c-MYC (**D**, brown) were also present on the media of the lesional vessels and on cells within the stroma (*short arrows*) of the *nidus*. Nuclei were counterstained with hematoxylin (**A–D**, blue). Original magnification: ×1,000.

ISH demonstrated the transcripts of OCT4 (Figure 4A), SOX2 (Figure 4B), KLF4 (Figure 4C), and c-MYC (Figure 4D) on the endothelium of the lesional vessels in all six AVM tissue samples. OCT4, KLF4, and c-MYC were also present on the media of lesional vessels and cells within the stroma of the *nidus*.

Positive controls showed transcript expression for OCT4 (Supplementary Figure 4A) on seminoma, SOX2 (Supplementary Figure 4B) on melanoma, KLF4 (Supplementary Figure 4C) on sweat glands, and c-MYC (Supplementary Figure 4D) on normal colon. The negative control, demonstrated on AVM tissue using a DapB probe, showed minimal expression (Supplementary Figure 4E).

### AVM-Derived Primary Cell Lines Expressed OCT4, SOX2, KLF4, and c-MYC Proteins

**Figure 5:**
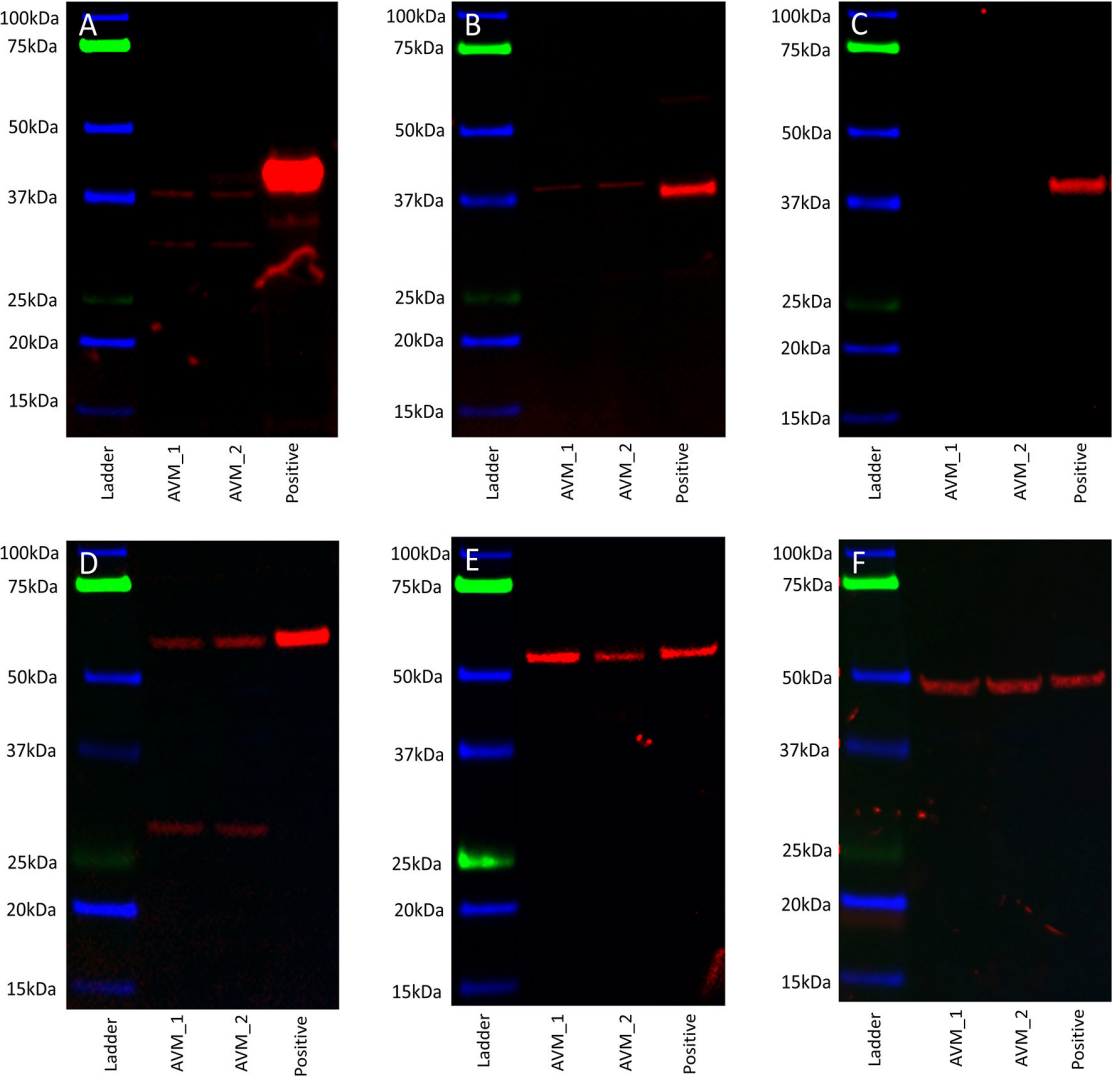
Representative western blot images of total protein extracted from two arteriovenous malformation-derived primary cell lines probed for OCT4 **(A)**, SOX2 **(B)**, NANOG **(C)**, KLF4 **(D)**, c-MYC **(E)**, and α-tubulin **(F)**, with appropriate molecular weights labeled.

WB performed on two AVM-derived primary cell lines demonstrated protein expression of OCT4 (Figure 5A, red) and SOX2 (Figure 5B, red) with bands at 40 kDa. NANOG was not detected (Figure 5C, red) in either cell line. KLF4 was detected at 54 kDa and 29 kDa (Figure 5D, red) and c-MYC was detected at 55 kDa (Figure 5E, red). Specificity was confirmed on the positive control (NTERA2 cell line). α-Tubulin loading control confirmed equal and consistent protein loading (Figure 5F).

### AVM-Derived Primary Cell Lines Expressed OCT4, SOX2, NANOG, KLF4, and c-MYC Transcripts

**Figure 6:**
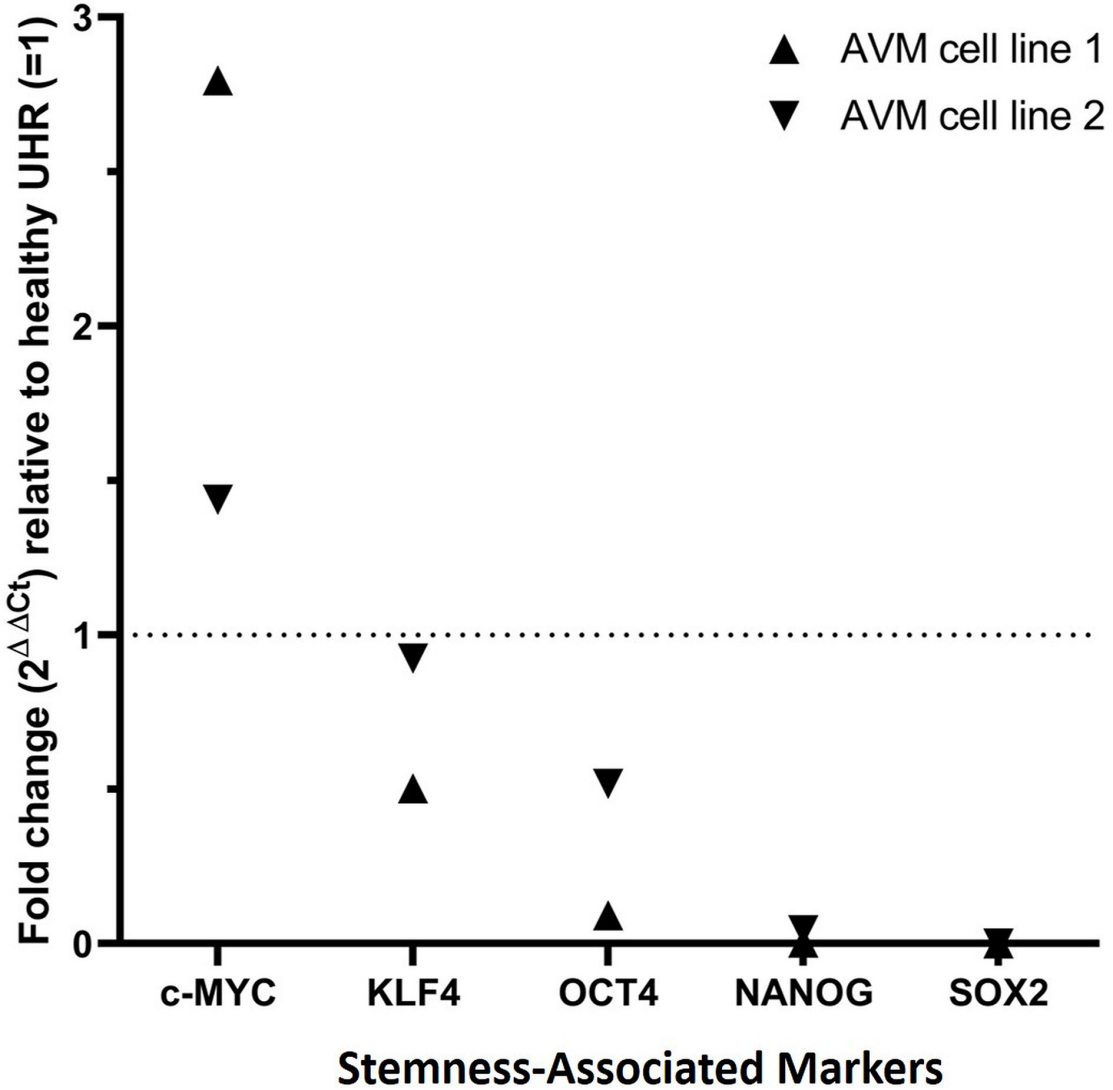
Graph of reverse transcription quantitative polymerase chain reaction performed on two arteriovenous malformation-derived primary cell lines, demonstrating mRNA expression of NANOG, SOX2, OCT4, c-MYC, and KLF4, normalized to the reference genes GAPDH, PSMB4, and PUM1 and expressed as fold change relative to UHR.

RT-qPCR performed on two AVM-derived primary cell lines confirmed mRNA expression of OCT4, SOX2, NANOG, KLF4, and c-MYC (Figure 6). The results compared the expression of the stemness-associated markers in AVM-derived cell lines, relative to the expression of the same markers within healthy UHR. c-MYC was the only marker with a higher expression in AVM-derived primary cell lines, of approximately 1.5- and 3-fold, relative to healthy UHR. KLF4 transcript expression was similar to healthy UHR. OCT4, followed by NANOG and SOX2, showed the least amounts of transcripts. NANOG and SOX2 showed minimal expression, relative to healthy UHR.

Electrophoresis of qPCR products on 2% agarose gels (Supplementary Figure 5) demonstrated specific amplification of the products. The expected size amplicons were observed, and no products were observed in the no template or no reverse transcriptase control reactions (Supplementary Figure 5).

## Discussion

In this study, immunohistochemical staining demonstrated the expression of the stemness-associated markers OCT4, SOX2, KLF4, and c-MYC but not NANOG on the endothelium and media of lesional vessels and cells within the stroma of the *nidus* in all 12 AVM tissue samples. This was confirmed by the transcript expression of OCT4, SOX2, KLF4, and c-MYC on ISH analysis of AVM tissue samples and by the protein expression on WB analysis of AVM-derived primary cell lines. RT-qPCR performed on the same cell lines detected all five stemness-associated markers, although NANOG and SOX2 showed minimal expression. Different combinations of the markers of interest were used during immunofluorescence staining, allowing us to efficiently investigate the co-expression within the tissues. Based on the co-expression of two markers on the same cells, we then assumed that in subsequent combinations where cells visibly expressed one of those markers, the other would also be expressed, as it was not feasible to co-stain for all five markers in one sample. This allowed us to deduce that all markers except NANOG were present and localized to the same areas within the vessels, demonstrating an OCT4+/SOX2+/KLF4+/c-MYC+ ESC-like population on the endothelium and media of lesional vessels and on cells within the stroma of the AVM *nidus*.

RT-qPCR showed the lowest expression of SOX2, contrasting with its distinct immunohistochemical and immunofluorescence staining and transcript expression on ISH analysis of AVM tissues. However, it has been suggested that transcript levels are not entirely predictive of protein expression (32). Discrepancies between transcript and protein expression levels, as seen with SOX2 in this study, have been previously observed (32–34). It has been attributed to several factors, such as the role of post-translational processing (32, 33) in the variability of protein levels relative to mRNA transcripts. The discrepancy has also been explained by the variable lengths of mRNA and protein half-lives (32, 34), the rate of translation, and its modulation by several factors (32) and by technical error which generates background noise (33, 34). c-MYC expression on immunohistochemical (Figure 2E) and immunofluorescence (Figures 3G,H) staining and WB (Figure 5E) aligned with abundant transcript expression on ISH (Figure 4D) and RT-qPCR analysis on AVM-derived primary cell lines (Figure 6). Interestingly, the oncoprotein c-MYC showed higher expression levels in AVM tissue than in healthy UHR. This supports the proliferative tendencies of AVM, suggesting a similar mechanism in cancer characterized by uncontrolled proliferation and dysregulated cellular processes, e.g., apoptosis and cell division (35). As KLF4 is associated with maintaining vascular integrity (25), its relatively high transcript and protein expression is consistent with the thickened vessels in AVM (8). Absent NANOG transcript expression aligns with the lack of NANOG protein expression on immunohistochemical (Figure 2C) and WB (Figure 5C) analysis.

Variable transcript expression is characteristic of iPSCs and ESCs, which display heterogeneous expression and temporal fluctuation of pluripotency genes including KLF4 and NANOG (36). OCT4, SOX2, and NANOG are the most primitive ESC markers (35), regulated by feedback loops (36) which allow downregulation and compensatory upregulation of other markers. This could explain the low SOX2 expression on RT-qPCR, alongside the relatively higher expression of NANOG and OCT4. NANOG was detected in the cell line samples using RT-qPCR, but not by WB. This may suggest transcriptional but not translational activation of NANOG. Although it is possible that this is due to the cell line constituents failing to accurately represent the composition of the tissues, the absence of NANOG on WB is unsurprising, as it has been proposed that NANOG is dispensable in induced pluripotency and the formation of iPSC under optimal cell culture conditions (24).

iPSCs are generated from somatic cells by overexpression of transcription factors OCT4, SOX2, NANOG, KLF4, and c-MYC, which are markers of iPSCs and facilitate the reprogramming of somatic cells back to a pluripotent state (37). The presence of the stemness-associated markers within lymphatic malformation (31) and AVM suggests the presence of an iPSC phenomenon which may be initiated and perpetuated by certain somatic mutations. A causative link between the genetic instability of iPSCs during reprogramming and an increased risk of disease has been proposed (37), with pre-existing mutations in somatic cells identified as the primary cause (15).

Somatic mutations have been identified in many types of vascular anomalies, including extracranial AVM (2, 15). It has been proposed that AVM results from a vascular developmental error during embryogenesis (3, 7, 38, 39), namely a somatic activating mutation in the MAP2K1 gene causing endothelial dysfunction through increased MEK1 activity (2).

Vaskova et al. (37) suggest that iPSCs often acquire genetic and epigenetic aberrations such as mutations during the reprogramming, when they display genomic instability and DNA damage (40). We speculate that somatic activating mutations may perpetuate a similar population underscoring the progression of AVM. The demonstration of cells in AVM that express stemness-associated markers may suggest the presence of ESC-like cells, and this warrants further investigation. The presence of such a primitive population would be consistent with the finding of ESC-like cells in other vascular anomalies such as infantile hemangioma (28), pyogenic granuloma (29), venous malformation (22), verrucous venous malformation (30), and lymphatic malformation (31).

This study demonstrated the presence of an OCT4+/SOX2+/KLF4+/c-MYC+ population on the endothelium and media of lesional vessels and on cells within the stroma of the AVM *nidus*. Further studies with a larger sample size including functional experiments are required to confirm our findings. Components of the renin–angiotensin system (RAS) have been shown to be expressed by similar populations in infantile hemangioma (41), pyogenic granuloma (42), and venous malformation (43, 44). Investigation into the expression of RAS on a primitive population in extracranial AVM may lead to novel targeting of the population using available medications.

## Data Availability Statement

The raw data supporting the conclusions of this article will be made available by the authors, without undue reservation.

## Ethics Statement

The studies involving human participants were reviewed and approved by Central Health and Disability Ethics Committee (Ref. 13/CEN/130). The patients/participants provided their written informed consent to participate in this study.

## Author Contributions

ST formulated the study hypothesis and design. CLK, PD, HB, and ST interpreted the immunohistochemical data. NB performed the confocal microscopy. CLK, PD, NB, and ST interpreted the immunofluorescence data. NB performed the WB analysis. NB, CLK, PD, and ST interpreted the WB data. JP performed the RT-qPCR experiments. JP, CLK, and ST interpreted the RT-qPCR data. EP performed the cell culture. CLK and ST drafted the manuscript. CLK, PD, and ST revised the manuscript. All authors commented on and approved the manuscript.

## Conflict of Interest

ST and PD are inventors of a provisional patent Treatment of Vascular Anomalies (PCT/NZ2017/050032), 2016; and Methods and compositions for the treatment of hemangioma (NZ761251), 2020. The remaining authors declare that the research was conducted in the absence of any commercial or financial relationships that could be construed as a potential conflict of interest.
