# Supplementary material for: Stemness-Associated Markers Are Expressed in Extracranial Arteriovenous Malformation
Source: Front Surg. 2021 Mar 19;8:621089. doi: 10.3389/fsurg.2021.621089 (PMC8017302; doi:10.3389/fsurg.2021.621089)
Supplement: Supplementary file 1 [file Data_Sheet_1.pdf]

Supplementary Material

Supplementary Table 1 Patient Demographics and Location of Their Arteriovenous Malformation

Patient	Age	Sex	Anatomical Location
1	22	F	Forehead/scalp
2	32	F	Lower lip/chin
3	65	F	Upper lip
4	54	F	Nose
5	19	M	Foot
6	44	F	Scalp/ear
7	17	F	Foot
8	48	M	Hand
9	17	M	Foot
10	20	M	Forearm
11	17	M	Foot
12	58	M	Hand

F, female; M, male

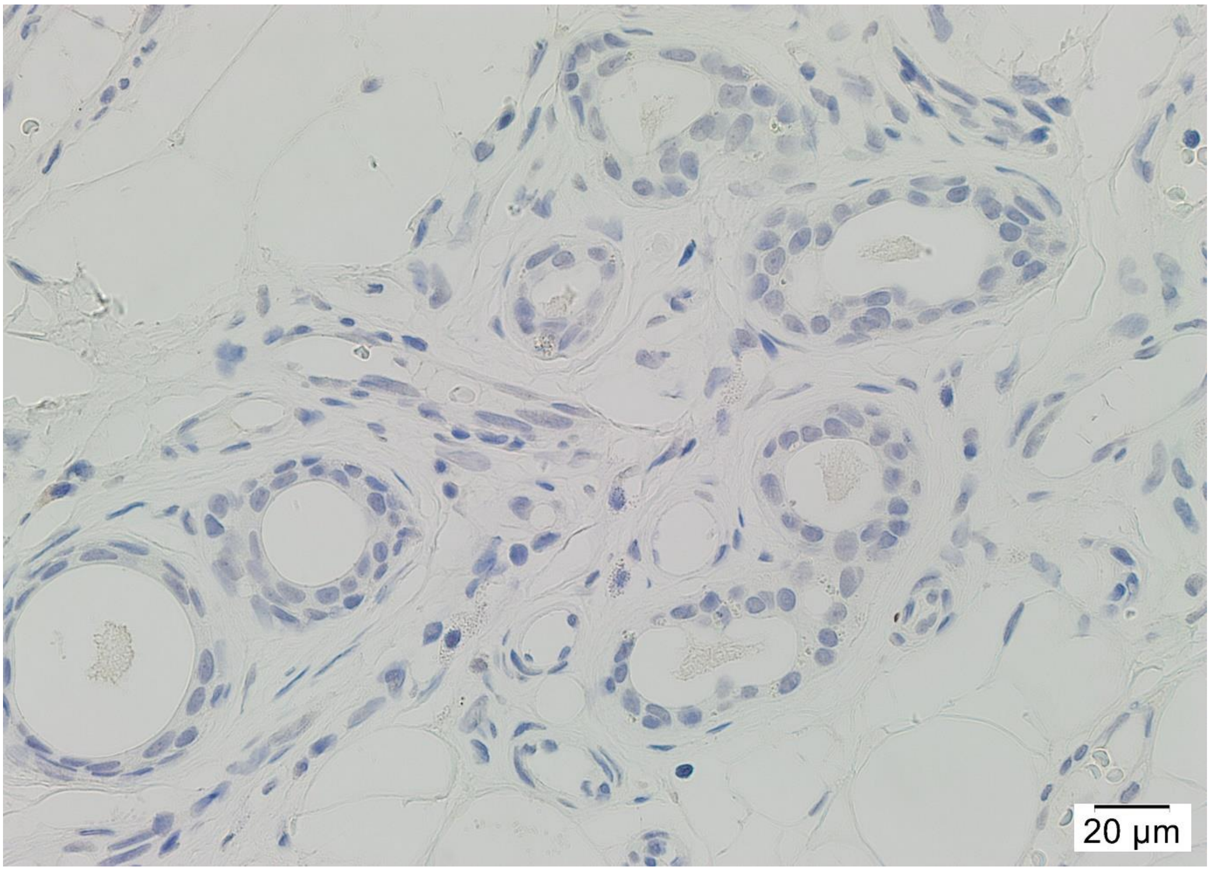

Supplementary Figure 1. Representative immunohistochemical-stained sections of arteriovenous malformation tissue samples, demonstrating no expression of NANOG (*brown*) on the *nidus*. Nuclei were counterstained with hematoxylin (blue). Original magnification: 400x.

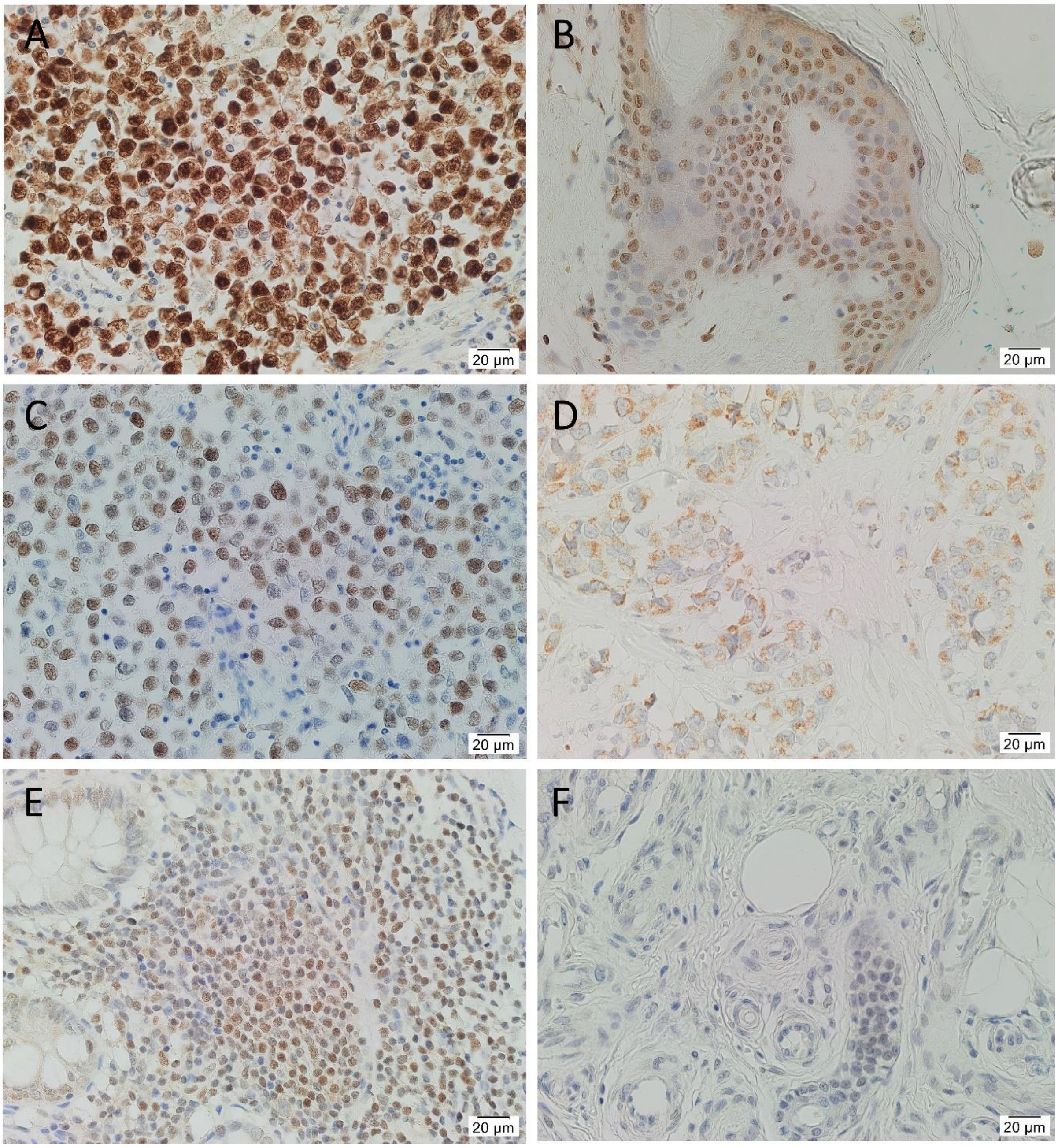

Supplementary Figure 2. Representative immunohistochemical-stained sections of human control tissues: seminoma for OCT4 (**A**, brown), skin (sweat glands) for SOX2 (**B**, brown), seminoma for NANOG (**C**, brown), breast carcinoma for KLF4 (**D**, brown) and normal colon for c-MYC (**E**, brown). Negative control performed on a section of arteriovenous malformation omitting the primary antibody showed no staining, confirming the specificity of the secondary antibody (**F**). Nuclei were counterstained with hematoxylin (**A-F**, blue). Original magnification: 400x.

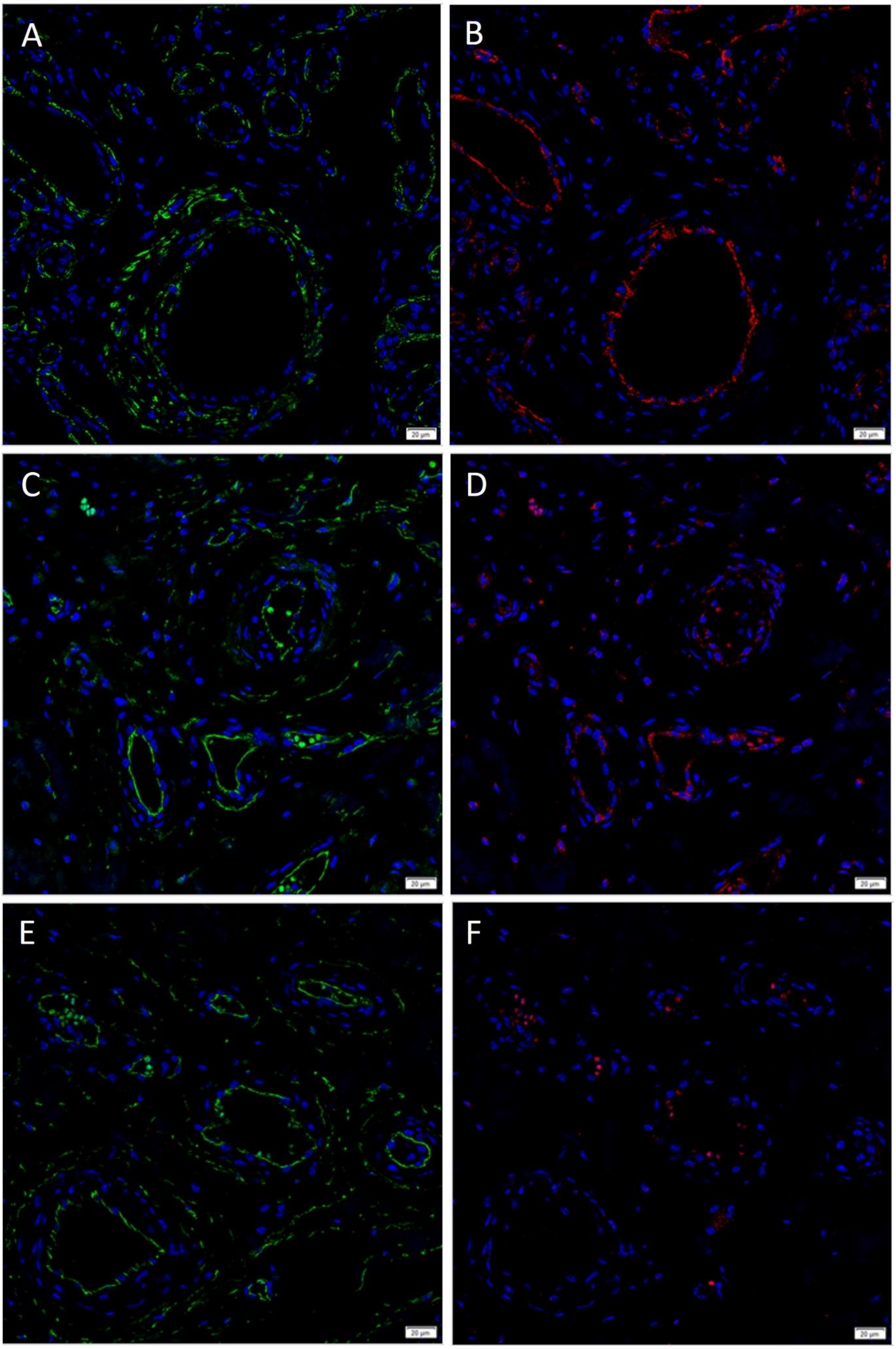
A
B
C
D
E
F

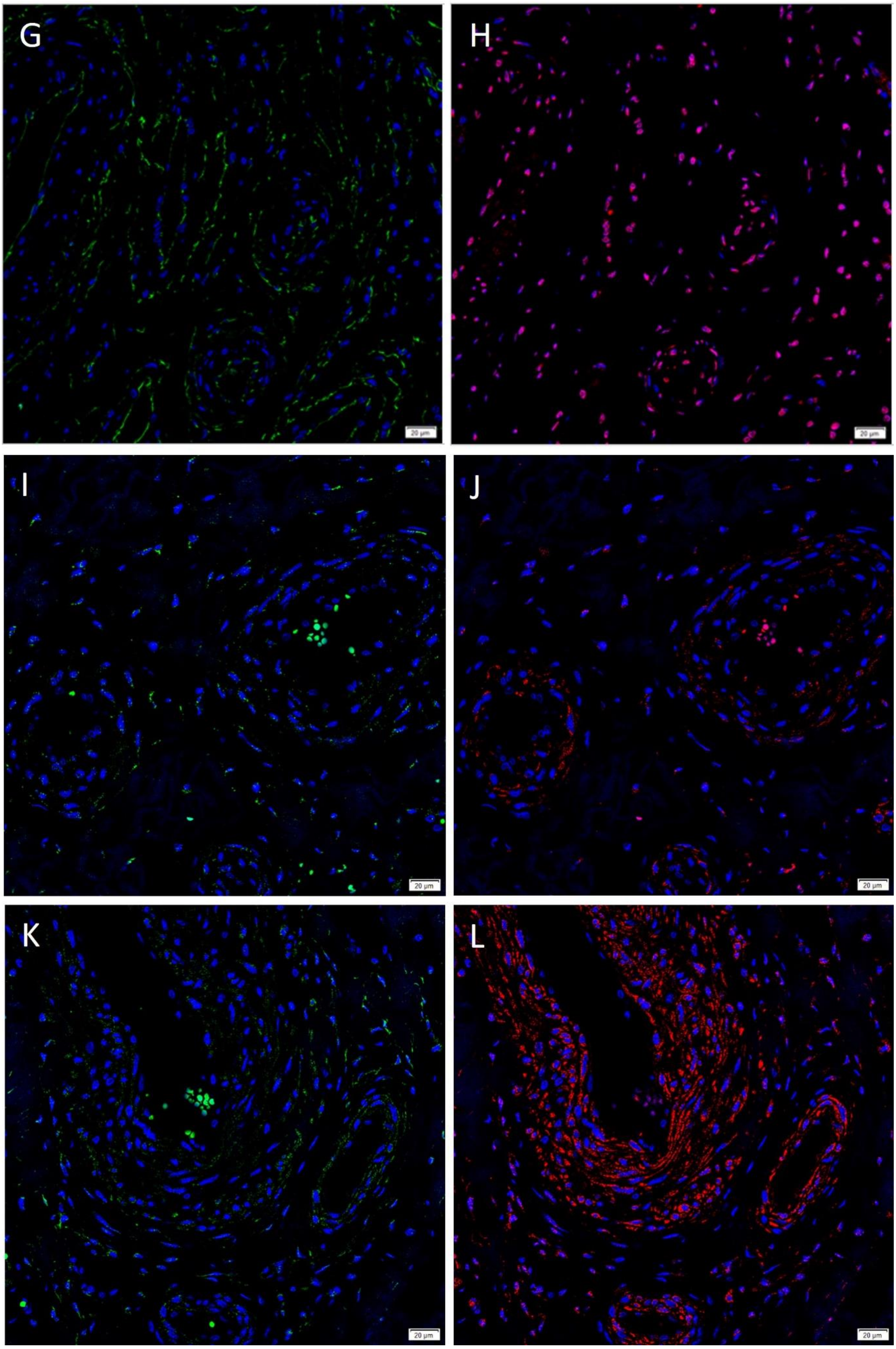
G
H
I
J
K
L
20 µm

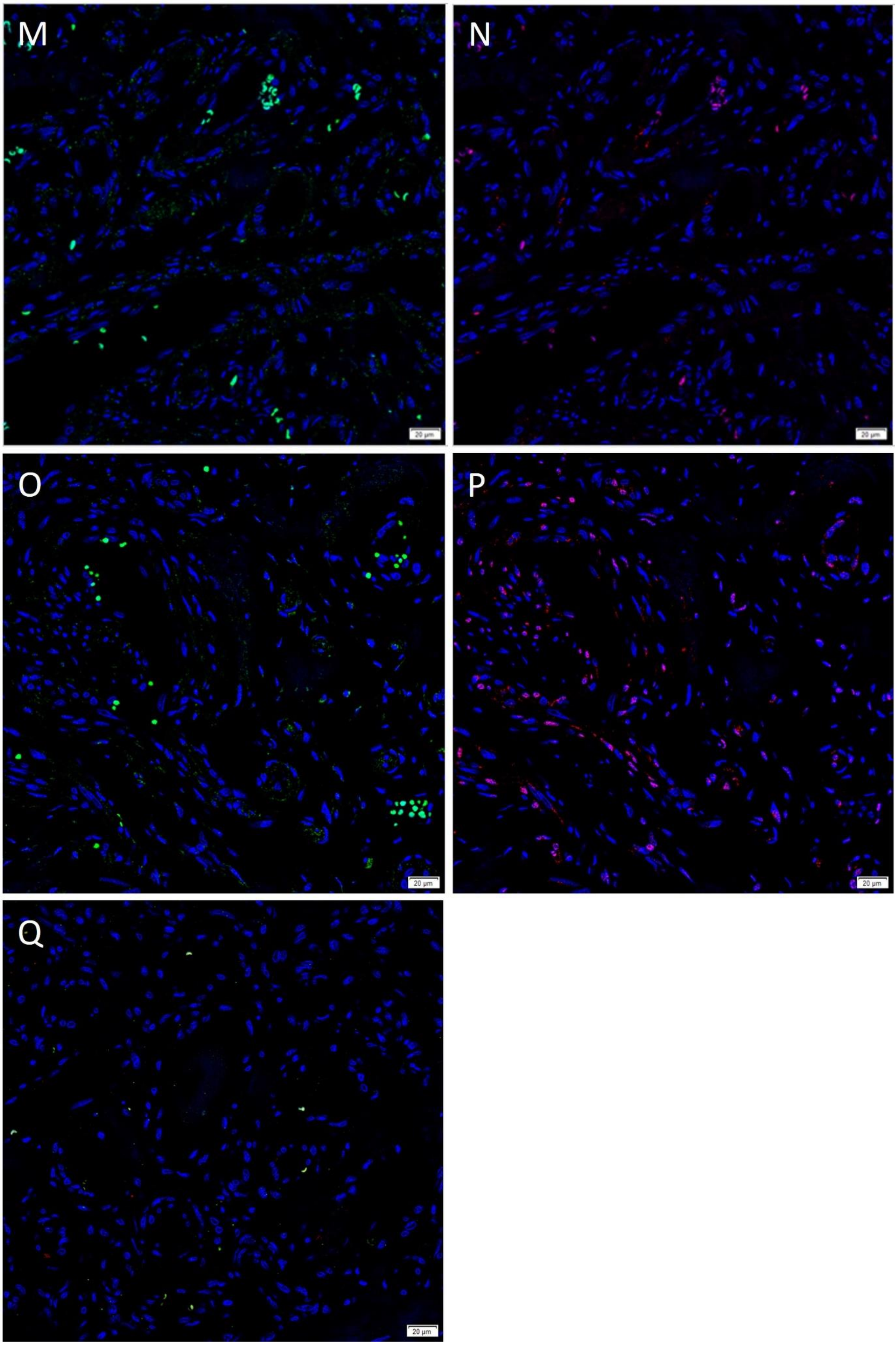
M
N
O
P
Q
20 µm

Supplementary Figure 3. Split images of immunofluorescence-stained sections of arteriovenous malformation tissue samples demonstrated in Figure 3, showing the expression of SMA (**A**, green) and vWF (**B**, red); OCT4 (**C**, red) and KLF4 (**D**, green); CD34 (**E**, green) and NANOG (**F**, red); CD34 (**G**, green) and SOX2 (**H**, red); OCT4 (**I**, green) and KLF4 (**J**, red); c-MYC (**K**, green) and KLF4 (**L**, red); OCT4 (**M**, green) and NANOG (**N**, red); c-MYC (**O**, green) and SOX2 (**P**, red). Negative control (**Q**) performed on a section of an arteriovenous malformation showed the specificity of the fluorescent secondary antibodies. Cell nuclei were counterstained with 4',6-diamidino-2-phenylindone (**A-Q**, blue). Original magnification: 400x.

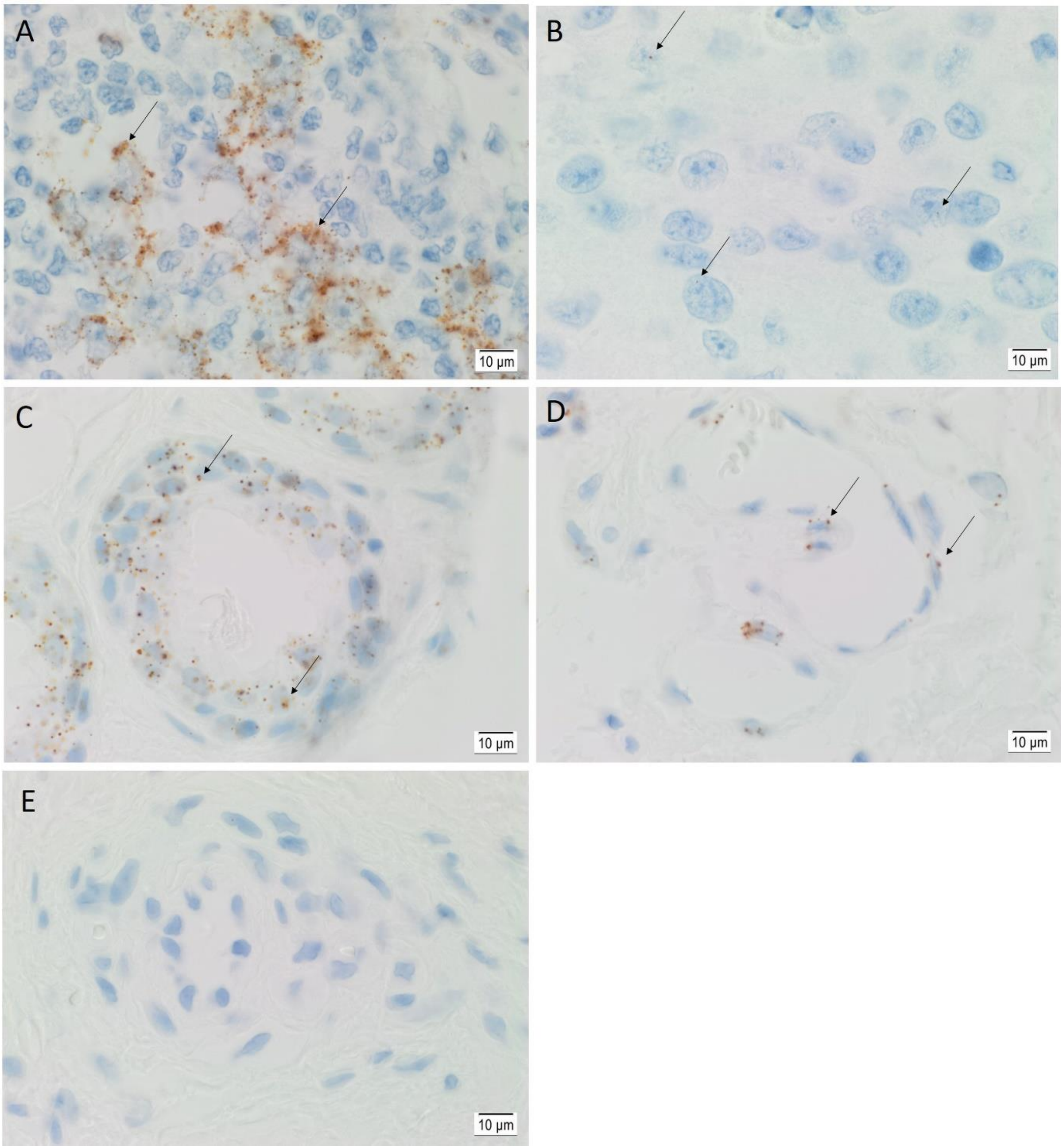

Supplementary Figure 4. Human positive control tissues for *in situ* hybridization: seminoma for OCT4 (**A**, brown, *arrows*), melanoma for SOX2 (**B**, brown, *arrows*), skin (sweat glands) for KLF4 (**C**, brown, *arrows*) and colon for c-MYC (**D**, brown, *arrows*). Background level of transcript was assessed with the negative control showing minimal staining (**E**). Nuclei were counterstained with hematoxylin (**A-E**, blue). Original magnification: 1000x.

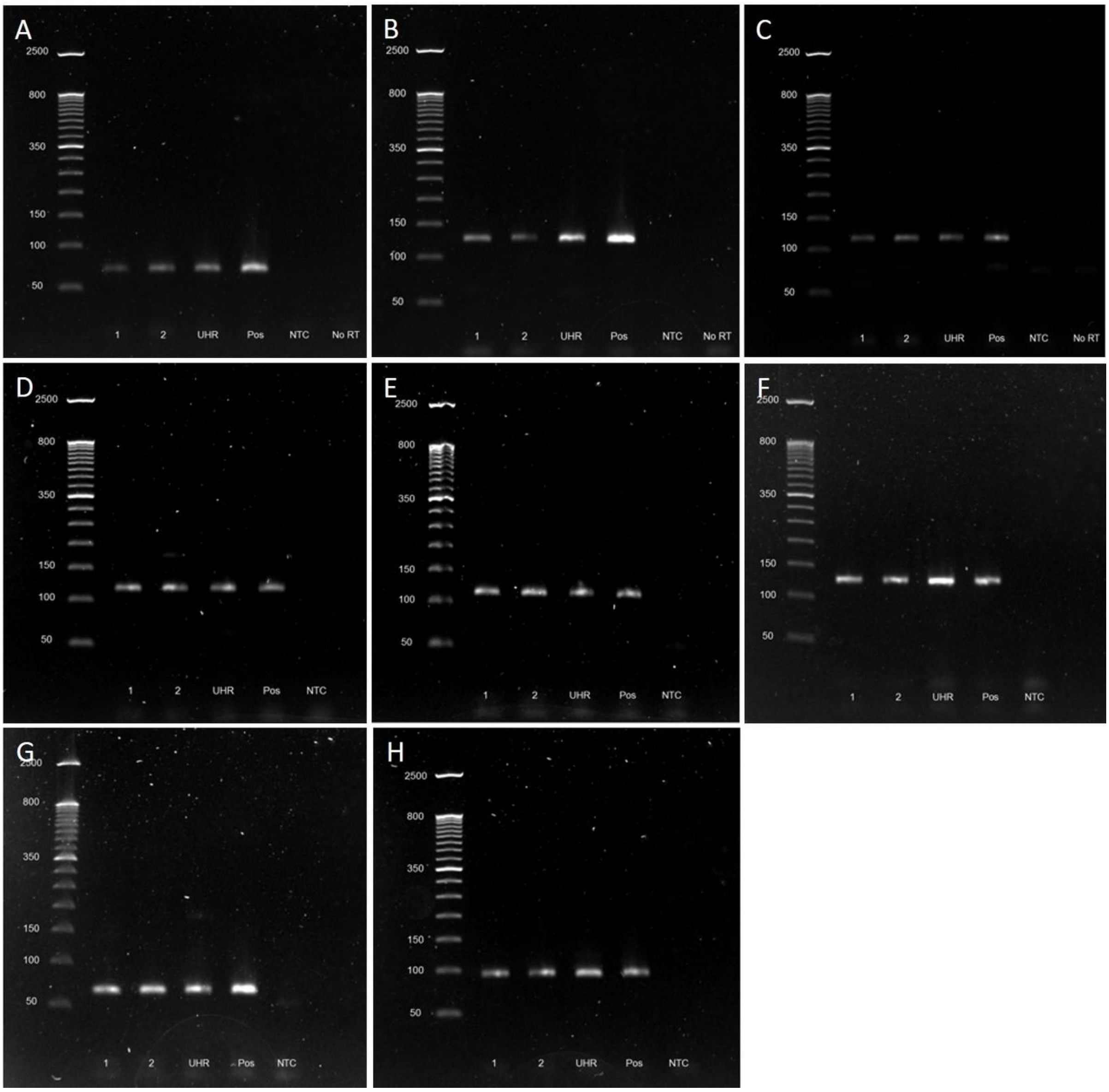

Supplementary Figure 5. Reverse transcription quantitative polymerase chain reaction amplification products from two arteriovenous malformation derived primary cell lines were checked using agarose gel electrophoresis. Probe specificity was confirmed for OCT4 (**A**, 64bp), NANOG (**B**, 109bp), SOX2 (**C**, 91bp), KLF4 (**D**, 110bp) and c-MYC (**E**, 107bp), and the reference genes GAPDH (**F**,122bp), PSMB4 (**G**, 63bp), and PUM1 (**H**, 63bp) were also checked. Ladder refers to the DNA marker in base pairs (bp); Lanes 1 and 2 refer to the respective cell lines; UHR, universal human RNA; Pos, positive control (NTERA2 cell lines); NTC, no template control (RNase-free water) to confirm no contamination; No RT, reverse transcription negative control for primers that may detect genomic DNA.
